# Solu: a cloud platform for real-time genomic pathogen surveillance

**DOI:** 10.1186/s12859-024-06005-z

**Published:** 2025-01-13

**Authors:** Timo Saratto, Kerkko Visuri, Jonatan Lehtinen, Irene Ortega-Sanz, Jacob L. Steenwyk, Samuel Sihvonen

**Affiliations:** 1 Solu Healthcare Oy, Kalevankatu 31 A 13, 00100 Helsinki, Finland; 2 Department of Food Technology, Safety and Health, Faculty of Bioscience Engineering, Ghent University, Coupure Links 653, 9000 Ghent, Belgium; 3 Howards Hughes Medical Institute and the Department of Molecular and Cell Biology, University of California, Berkeley, Berkeley, CA USA

**Keywords:** Workflow, Genomics, Whole-genome sequencing, Infection prevention, Outbreak, Phylogeny, Privacy

## Abstract

**Background:**

Genomic surveillance is extensively used for tracking public health outbreaks and healthcare-associated pathogens. Despite advancements in bioinformatics pipelines, there are still significant challenges in terms of infrastructure, expertise, and security when it comes to continuous surveillance. The existing pipelines often require the user to set up and manage their own infrastructure and are not designed for continuous surveillance that demands integration of new and regularly generated sequencing data with previous analyses. Additionally, academic projects often do not meet the privacy requirements of healthcare providers.

**Results:**

We present Solu, a cloud-based platform that integrates genomic data into a real-time, privacy-focused surveillance system.

**Evaluation:**

Solu’s accuracy for taxonomy assignment, antimicrobial resistance genes, and phylogenetics was comparable to established pathogen surveillance pipelines. In some cases, Solu identified antimicrobial resistance genes that were previously undetected. Together, these findings demonstrate the efficacy of our platform.

**Conclusions:**

By enabling reliable, user-friendly, and privacy-focused genomic surveillance, Solu has the potential to bridge the gap between cutting-edge research and practical, widespread application in healthcare settings. The platform is available for free academic use at https://platform.solugenomics.com.

**Supplementary Information:**

The online version contains supplementary material available at 10.1186/s12859-024-06005-z.

## Background

Bacterial and fungal pathogens, along with their antimicrobial resistance, are causing an increasing burden on healthcare and public health [1–3]. Advances in microbial genomics have significantly enhanced infection prevention and outbreak surveillance by providing detailed information about pathogen species, antimicrobial resistance, and phylogenetics [4, 5]. As the cost of Whole-Genome Sequencing (WGS) has decreased rapidly, continuous genomic surveillance has become a cost-effective method for infection prevention and control [6, 7]. The interest towards genomic analysis has led to the emergence of several pathogen analysis tools, such as nf-core [8], TheiaProk [9], ASA3P [10], CamPype [11], Nullarbor [12], Bactopia [13], and Galaxy [14], which enable genomic analysis also for users without in-depth expertise in bioinformatics or computer science.

Despite these advancements, bioinformatics still remains a bottleneck for the widespread adoption of pathogen genomic surveillance due to limitations in usability, speed, and security [7].

Most existing pipelines [8–13] are operated using the command-line interface (CLI) and require the user to manage their own data storage and computation infrastructure. While it is possible to learn their usage without advanced computational knowledge [15], many practitioners simply don’t have the time or willingness for it and prefer graphical user interfaces instead. Additionally, most existing tools are designed for single-use execution, which is a challenge for continuous surveillance, where new sequencing data is often generated in small batches [6, 16]. To facilitate ongoing analysis, users must implement their own processes for integrating new and old data.

Fast time to results has been identified as a key component for effective genomic surveillance [6, 16]. As new samples arrive in batches and need to be compared to all previously accumulated samples, computation time can become a significant bottleneck if using a single-workstation installation. Also, unless the pipelines are highly automated, running the analyses often requires specially trained personnel who might not be available immediately upon the arrival of new data.

Academia-led projects developed under FAIR (Findable, Accessible, Interoperable, Reusable) principles often lack the necessary privacy focus to meet the stringent requirements of healthcare providers [17]. In contrast, healthcare providers must adhere to stringent legal requirements, such as the U.S. HIPAA Privacy Rule [18] or the ISO27001 standard [19], ruling out many existing online platforms for genomic surveillance.

It is possible for healthcare providers to overcome these limitations by implementing their own automated pipelines, but it requires significant investments in bioinformatics and computational infrastructure, and the lack of these resources is a challenge in many facilities [20]. To fill this gap, we present Solu—an automated, fast, and secure web application for analyzing WGS samples.

## Implementation

Solu is a cloud-based platform for the analysis of bacterial and fungal WGS samples. Its automated bioinformatics pipeline includes genomic characterization and phylogenetic comparison. Its cloud implementation is built to match the usability, speed, and security requirements of ongoing genomic surveillance in healthcare facilities.

### Bioinformatics pipeline

**Fig. 1:**
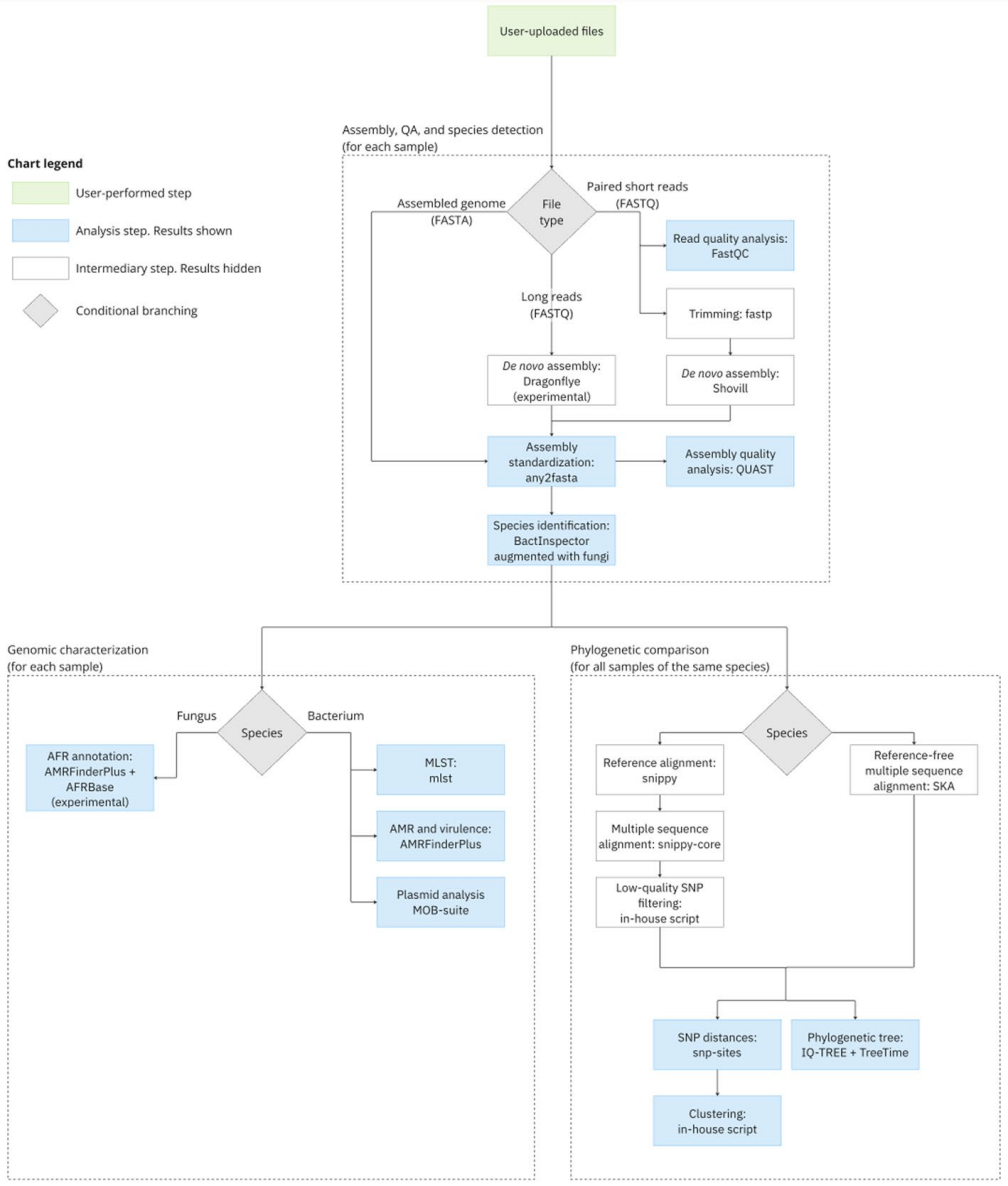
Bioinformatics pipeline

The platform runs a fully automated pathogen analysis pipeline, which is illustrated in Fig. 1. The pipeline includes de novo assembly, quality assurance (QA), species identification and genomic characterization for each uploaded sample, and phylogenetic comparison between all uploaded samples of the same species. It is triggered automatically after each file upload and cannot be configured by the user. This section presents an overview of the pipeline, and a detailed description can be found in Additional file 1.

#### Input format

The supports three input types: paired-end short reads in FASTQ format, long reads in FASTQ format, or an assembled genome in FASTA format. Analysis of long reads is still considered an experimental feature.

#### Assembly, QA, and species detection

Short reads are quality checked using FastQC [21], quality corrected with fastp [22], and assembled using Shovill [23]. Long reads are pre-processed, assembled and polished with Dragonflye [24]. After assembly, all samples are standardized using any2fasta [25] and quality assessed with Quast [26].

Species is identified with Bactinspector [27]. To identify fungal species, Bactinspector’s default database was augmented with all fungal reference genomes from the NCBI Taxonomy [28]. The augmented database also includes clade-level reference genomes for *Candida auris*.

#### Genomic characterization

Analysis of bacterial species includes multi-locus sequence typing (MLST) with mlst [29], AMR annotation using AMRFinderPlus [30], and plasmid analysis with MOB-suite [31].

The pipeline also includes an experimental antifungal resistance (AFR) gene annotation for the species *Candida auris*. AFR annotation is implemented using AMRFinderPlus with a custom database of known AFR point mutations sourced from AFRBase [32].

#### Phylogenetic comparison

The pipeline’s phylogeny is based on constructing a multiple sequence alignment for each species. Based on the species in question, multiple sequence alignment is computed by either a reference-based or reference-free method.

The reference-based alignment is considered more robust and has been implemented for 21 commonly analyzed species. It includes aligning each sample to the species’ reference genome using Snippy [33], creating a multiple-sequence alignment using snippy-core [33], and filtering out low quality SNPs using an in-house script.

The reference-free alternative is implemented to support analysis of species that are not yet supported by the reference-based alignment. It is computed using the split-kmer analysis tool SKA [34].

After constructing the multiple sequencing alignment, the phylogenetic comparison includes pairwise SNP distances, clustering, and phylogenetic tree inference. SNP distances are counted from the multiple sequence alignment using snp-sites [35] and snp-dists [36]. Samples are clustered with a 20-SNP single-linkage clustering threshold using an in-house Python script. Phylogenetic trees are inferred using a general time reversible maximum likelihood model from IQ-TREE 2 [37] and midpoint-rooted using TreeTime [38].

### Automated cloud infrastructure

The cloud infrastructure of Solu is built on three principles: usability, speed, and security.

#### Usability

**Fig. 2:**
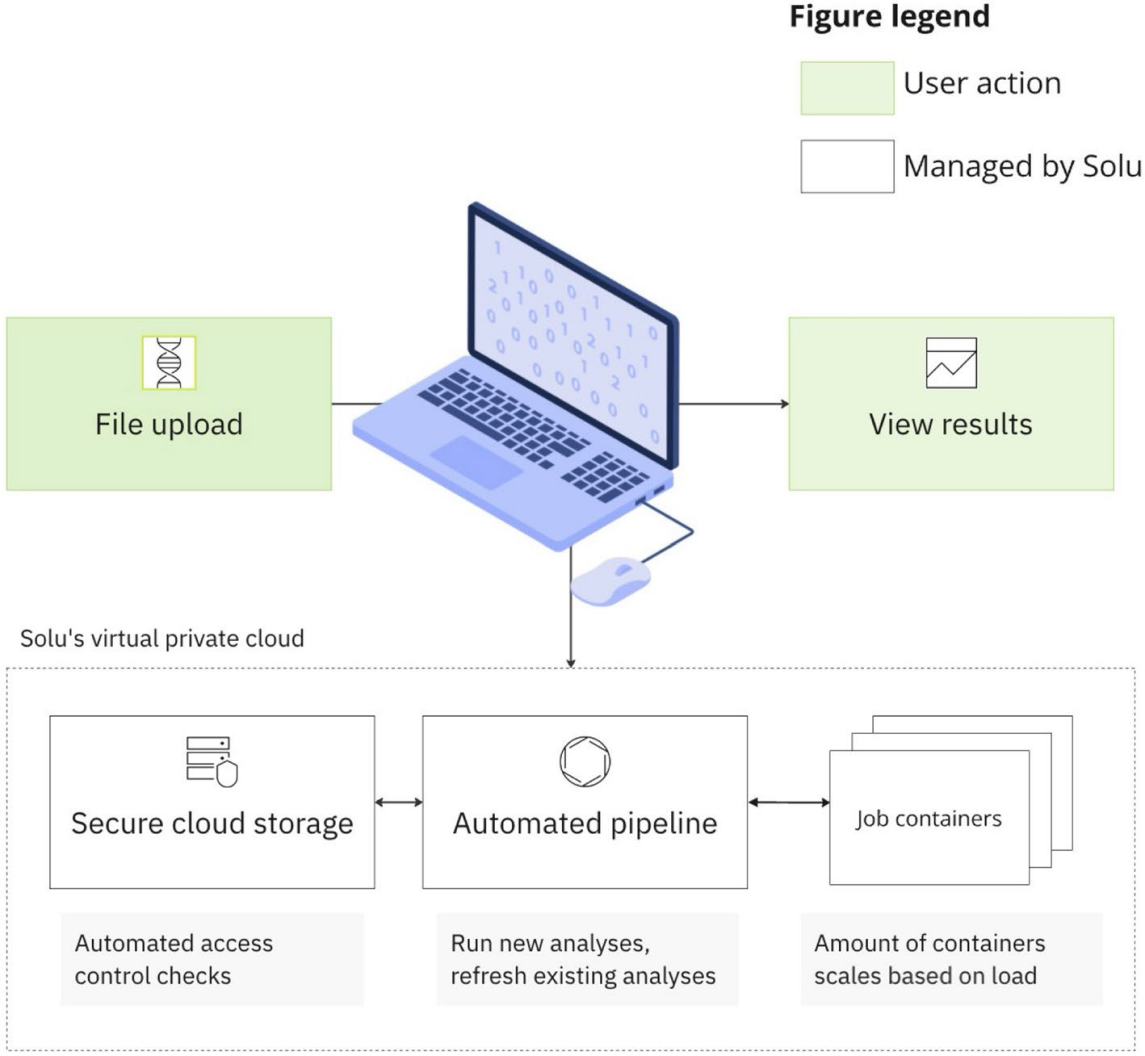
Solu’s cloud platform implementation

The Solu Platform is web based, enabling practitioners to use it without installing software or running command-line tools (Fig. 2). New samples are uploaded using the drag-and-drop web UI, which automatically triggers a bioinformatics pipeline. The pipeline requires zero configuration from the user, which promotes repeatability and alleviates the need for in-depth bioinformatics knowledge. The analysis results are stored in the cloud, eliminating the need for a self-implemented storage system and enabling effortless result sharing between colleagues.

Each newly uploaded sample is also automatically compared to previously uploaded samples of the same species, which enables detecting potential new outbreaks quickly. For instance, when a user uploads a *Salmonella enterica* sample, the platform automatically re-computes the phylogenetics for all uploaded *Salmonella enterica* samples and highlight possible clusters.

#### Speed

The platform’s cloud infrastructure is optimized for speed even during peak usage. This is achieved by running each computation-intensive workload in a separate Docker container with optimized resource (CPU and memory) distribution. These containers are orchestrated by a cloud computing cluster that is automatically scaled up and down based on usage, up to a maximum of 512 CPUs and 2 TB of memory. The cluster also contains a pool of hot standby resources, which allows starting the analysis of a new sample within seconds of its upload.

This auto-scaling capability brings the user substantial speed improvements by allowing the parallelization of some of the analyses in the pipeline. In addition, it allows analyzing a whole batch of samples simultaneously, leading to a significant reduction in overall time-to-results when analyzing a batch of samples. Importantly, these speed improvements are achieved without a significant increase in computation costs.

#### Security

The platform’s data is stored in a secure cloud storage with set read and write permissions. All computations occur within a virtual private network, monitored by automated access control checks. Solu implements strict data security protocols, including appropriate access permissions, encryption, continuous monitoring, code reviews, staff training, and other cybersecurity measures. Accordingly, Solu adheres to the ISO27001 security standard and to the U.S. HIPAA rule and can sign a Business Associate Agreement (BAA) for enterprise customers. Solu also allows enterprise customers to choose between U.S. or EU as their data storage. Further information regarding data security practices can be found at https://solugenomics.com/trust.

### Evaluation

To evaluate the Solu platform, we reproduced four outbreak investigation studies using published genomic data (Table 1). Data was obtained from the European Nucleotide Archive as raw reads and uploaded to the Solu platform. All samples were paired-end short-reads in FASTQ format.

**Table 1:** Overview of evaluation datasets

Species	BioProject accession	Number of samples	Raw data size (GB, zipped)
*Staphylococcus aureus*	PRJNA400143 [40]	135	16.47
*Enterococcus faecium*	PRJEB34664 [41]	99	15.18
*Salmonella enterica*	Multiple [42]	23	5.03
*Candida auris*	PRJNA328792 [43]	47	50.94

We evaluated Solu's performance by computing metric scores for species identification, MLST, clade construction, and AMR predictions against the references. Phylogenetic trees were exported from Solu. Tree topologies were compared visually, and where raw tree data was available, we calculated a Robinson-Foulds distance using TreeDist [39]. Both reference-based and reference-free phylogenetic pipelines were run for all datasets and compared against each other to validate the platform’s internal consistency.

We also measured the required time for analysis of each sample. Plasmids were not evaluated in this study due to the absence of plasmid annotations in the original studies.

## Results

### Evaluation results

**Fig. 3:**
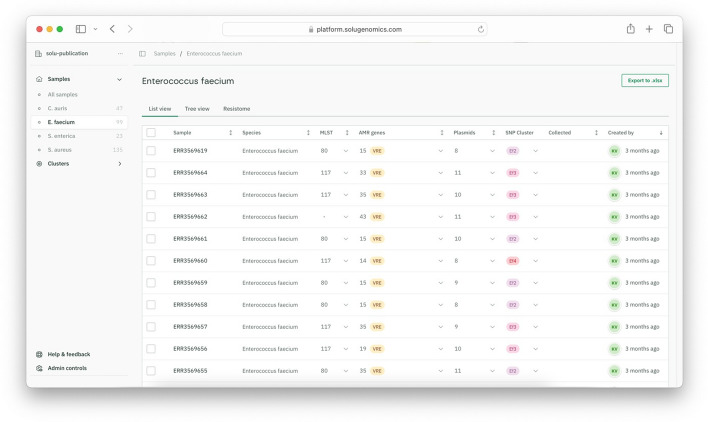
Summary of the samples shown in Solu

The Solu platform successfully completed the bioinformatics pipeline for all 304 samples. A screenshot of the platform’s home screen is shown in Fig. 3. This workspace, including all samples and results, is also accessible at a user-friendly web interface at https://platform.solugenomics.com/w/solu-publication.

#### Species, MLST and clade assignment

Solu accurately identified the species of all 304 samples and assigned the correct clade for all 47 *Candida auris* samples.

Exact MLST matches were observed in 210 out of 230 (91.3%) isolates with known sequence types (Table 2). However, 18 of 20 non-exact MSLT matches were single-locus variants.

**Table 2:** Species identification and MLST concordance. Solu’s results compared to the original publications when available there

Species	Species identification accuracy	MLST exact match accuracy	MLST accuracy including single-locus variants
*S. aureus*	100.0% (135/135)	87.1% (115/132)	98.4% (130/132)
*E. faecium*	100.0% (99/99)	96.9% (95/98)	100.0% (98/98)
*S. enterica*	100.0% (23/23)	*MLST not reported in the original article*	
*C. auris*	100.0% (47/47)	*MLST scheme not defined*	

#### Antimicrobial resistance

The antimicrobial resistance (AMR) gene detection results from Solu were compared with those of the references. Concordance varied by species, ranging from 99.6% for *E. faecium* to 93.1% for *S. aureus*. Table 3 summarizes some commonly studied AMR loci, while the full results can be viewed online.

**Table 3:** Sensitivity of Solu’s AMR prediction

Species	Overall AMR locus sensitivity	*vanA*	*vanB*	*mecA*	Other key genes
*S. aureus*	93.1% (309/332)	N/A	N/A	98.8% (81/82)	*mupA*: 100% (3/3), *blaZ*: 90.9% (100/110)
*E. faecium*	99.6% (515/517)	100.0% (30/30)	100.0% (69/69)	N/A	*ermB*:100.0% (95/95) *tet*: 100.0% (4/4)
*S. enterica*	*AMR results not reported in the original article*				
*C. auris*	90.9% (40/44)	N/A	N/A	N/A	*ERG11_K143R*: 100.0% (8/8)

Note: N/A indicates that the gene is not typically relevant for the species

Key AMR genes, such as the *vanA/vanB* type and *mecA*, were detected with 100% and 98.8% sensitivity, respectively. Antifungal resistance mutation detection for Candida auris showed a 90.9% sensitivity. The results matched the original findings for 43 isolates. However, Solu identified the *ERG11_K143R* mutation in 2 Clade I isolates, which were originally reported as having the *Y123F* mutation, and detected 2 isolates lacking ERG mutations.

The main reason for lower agreement in the *S. aureus* dataset was Solu's inability to find any *dfrA* matches, whereas the original article reported 10 isolates with *dfrA*.

#### Phylogenetics

**Fig. 4:**
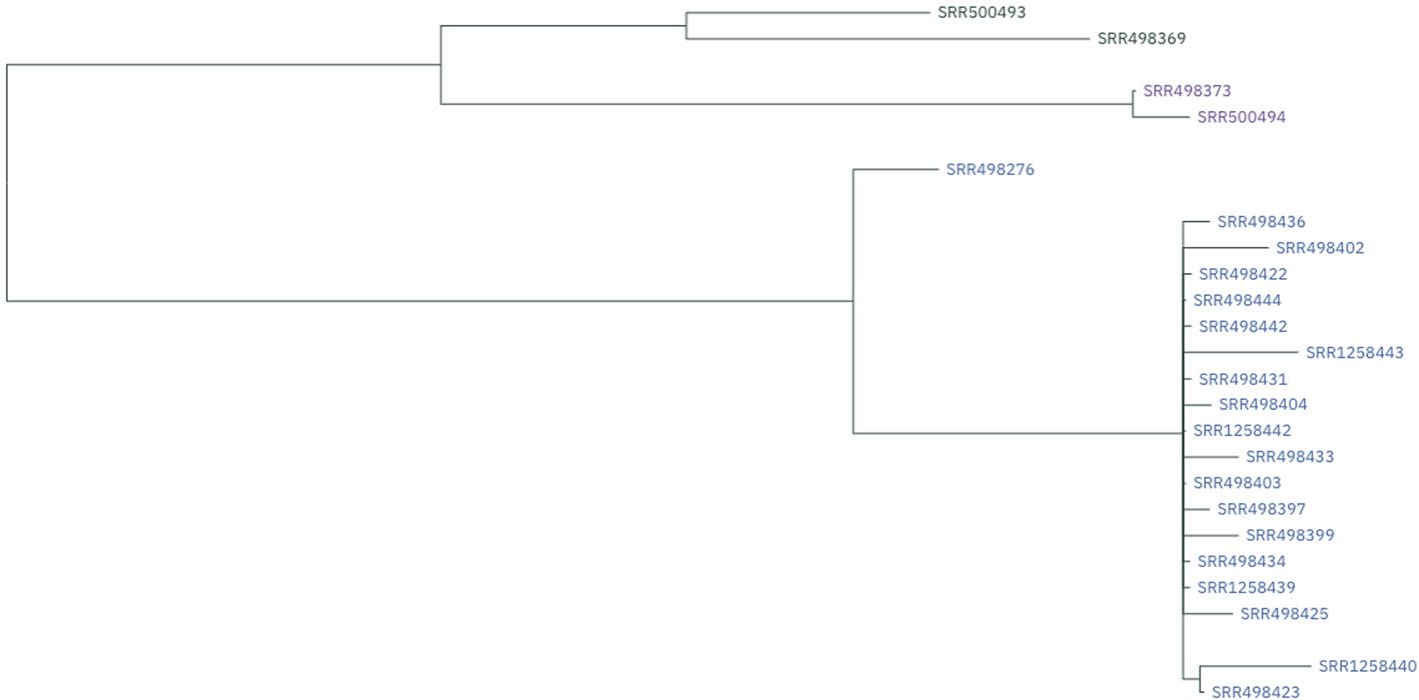
Screenshot of the *Salmonella enterica* tree in the graphical user interface

Solu automatically generated phylogenetic trees for all four datasets, which can be viewed and downloaded in the published workspace in “Tree view” (Fig. 4).

**Fig. 5:**
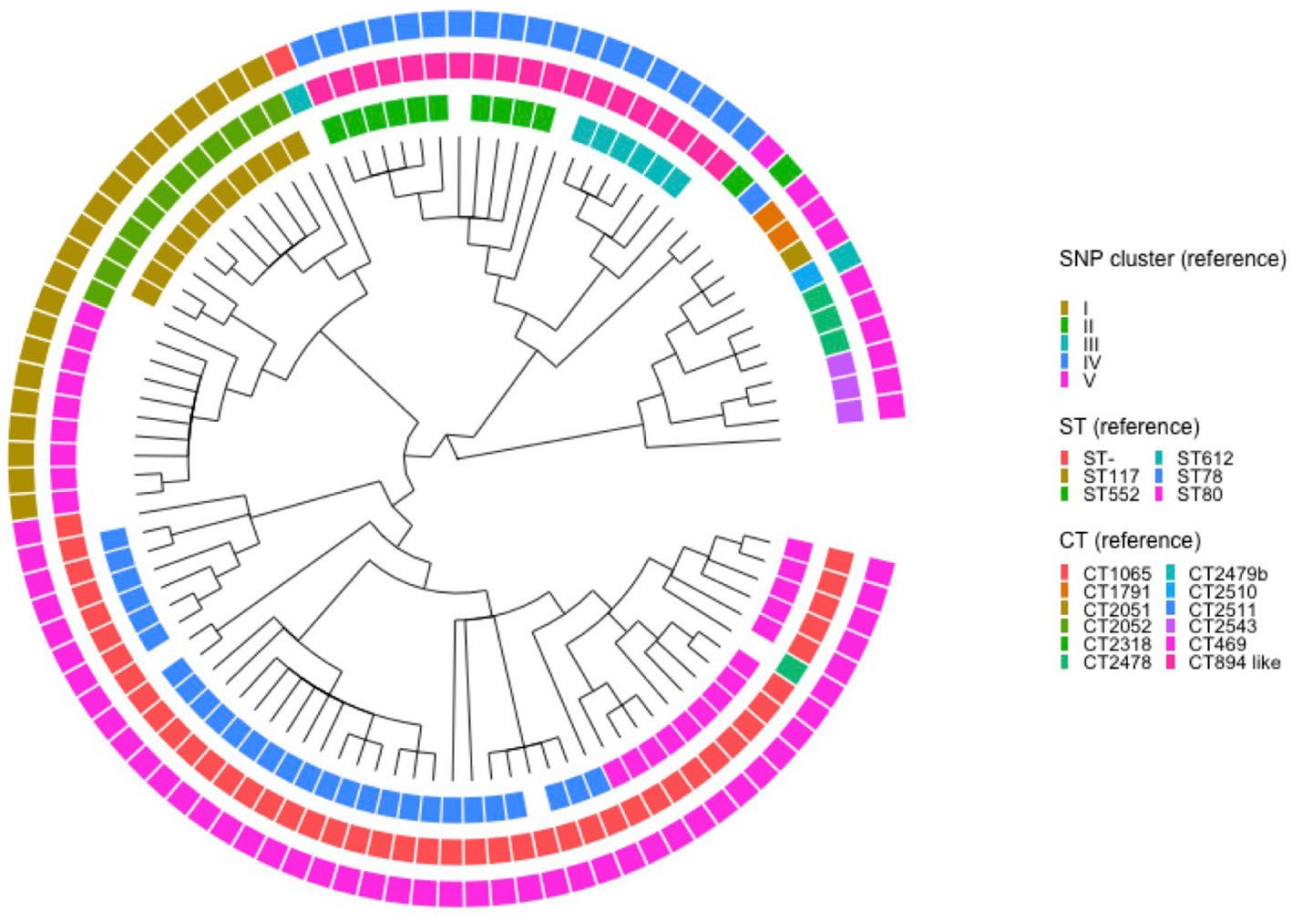
Solu’s *E. faecium* phylogenetic tree shown as a cladogram (inner) vs. SNP clusters, complex types (CT), and sequence types (ST) from the original publication (outer rings)

**Fig. 6:**
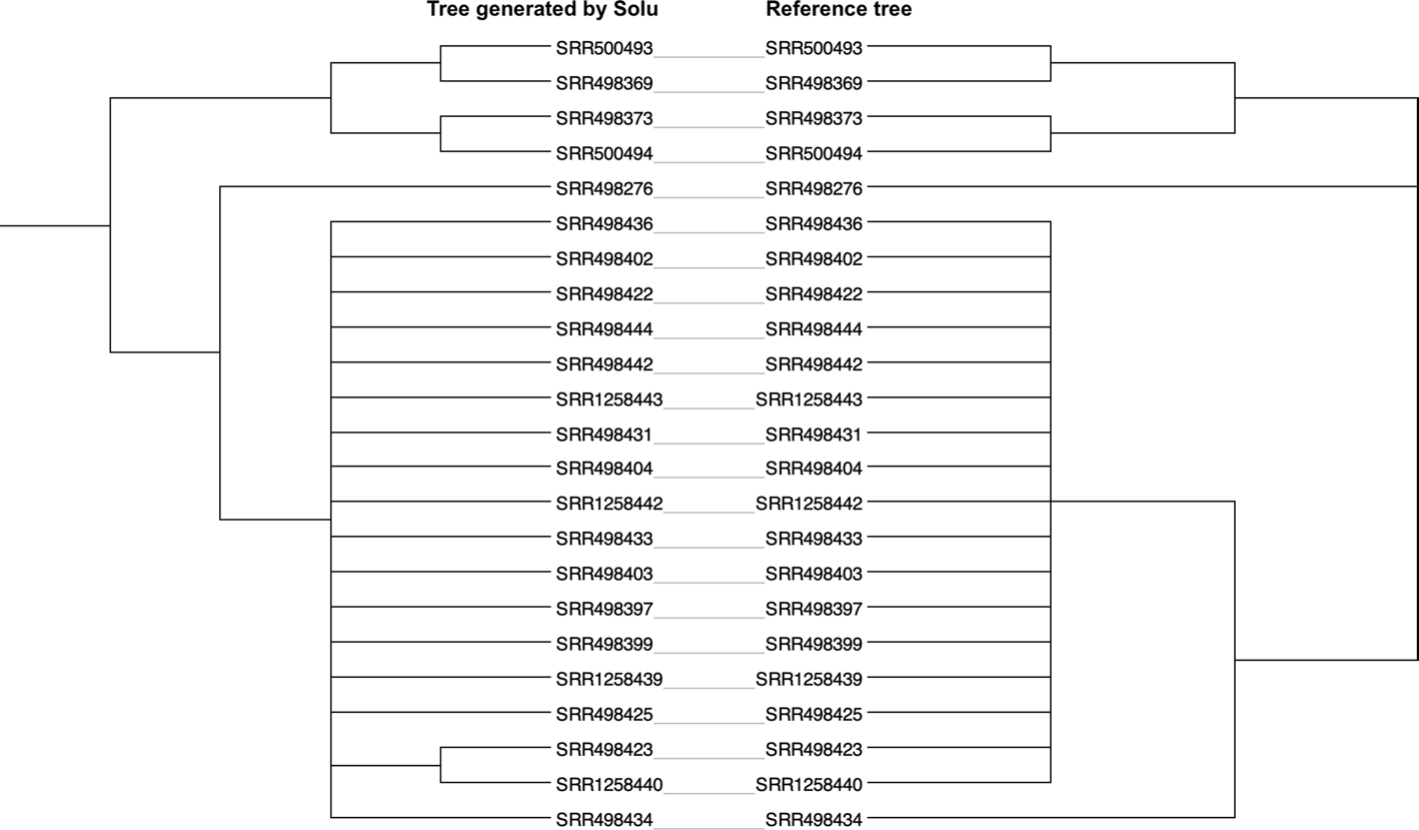
Tanglegram of Solu’s *Salmonella enterica* phylogenetic tree (left) vs. the reference tree (right). The tanglegram visualization was created using Dendroscope [44]

The *E. faecium* phylogenetic tree generated by Solu demonstrated a high degree of concordance with the reported SNP subclusters, complex types (CTs) and sequence types (STs) of the reference (Fig. 5). For the *S. enterica* dataset, Solu produced a similar topology to the reference tree (Fig. 6) where the outbreak samples are separate from the outgroup. Robinson-Foulds distance to the *S. enterica* reference tree was 2.

For the *S. aureus* and *C. auris* datasets, Solu generated phylogenetic trees in which isolates with identical sequence types or clades consistently clustered together. Further detailed comparisons were not possible due to the lack of raw tree data and subtyping information. The resulting trees are provided in Additional file 2.

In comparing the reference-free and reference-based pipelines, Solu’s reference-free pipeline generated highly concordant phylogenetic trees with the reference-based pipeline. The Robinson-Foulds distances ranged from 0.08 to 0.46, as computed using TreeDist [45], indicating a high level of similarity (see Additional file 2).

### Time-to-results

**Fig. 7:**
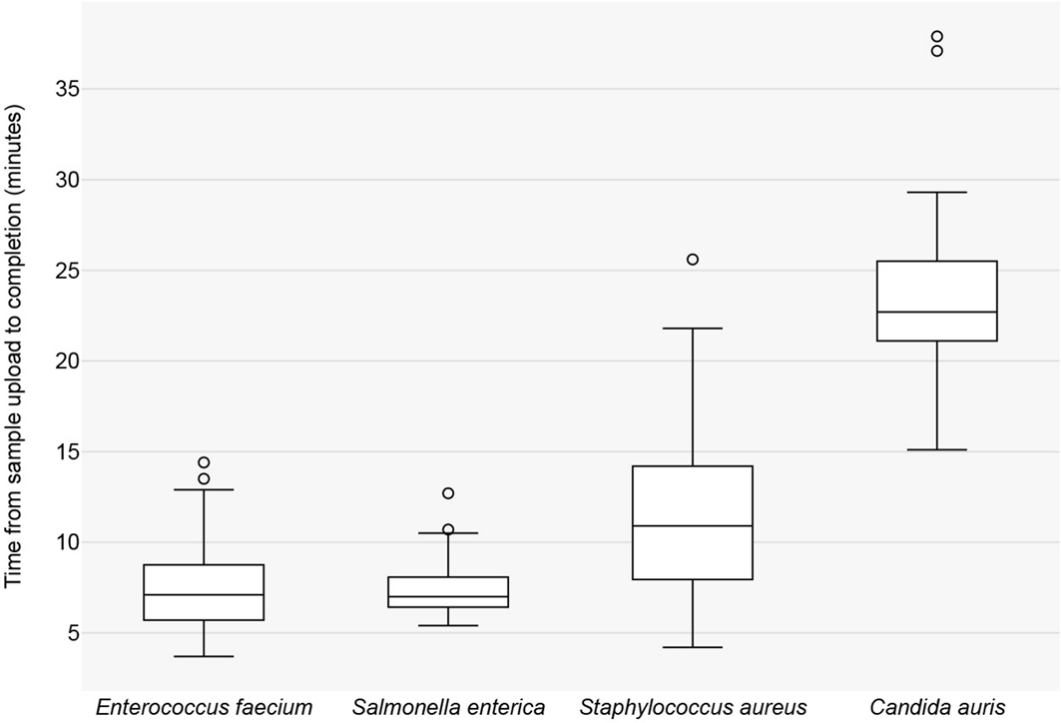
Box plot of Solu’s total time-to-results for each dataset

Time-to-results for the four datasets are presented in Table 4 and Fig. 7. For bacterial samples, Solu completed de novo assembly in an average of 7.2 min and variant calling in 9.5 min from upload. For *C. auris* samples, de novo assembly averaged 17 min, and variant calling took 23.6 min from upload.

**Table 4:** Average time-to-results per dataset**.** Shortest and longest recorded times in parentheses. Genome size [28] and sample count of each dataset included for additional context

			Average total time from sample upload (minutes)		
	Genome size (Mb)	Sample count	Read quality analysis and correction	De novo assembly	Variant calling
*Enterococcus faecium*	2.9	99	1.5 (1.0–2.8)	5.2 (1.9–11.8)	7.4 (3.7–14.4)
*Salmonella enterica*	5.0	23	1.4 (1.1–1.8)	5.0 (2.9–10.1)	7.6 (5.4–12.7)
*Staphylococcus aureus*	2.8	135	1.9 (1.0–6.1)	9.0 (2.4–21.9)	11.4 (4.2–25.6)
*Candida auris*	12.2	47	3.5 (2.4–5.9)	17.0 (10.4–32.3)	23.6 (15.1–37.9)

## Discussion

Bioinformatics remains a bottleneck to widespread use of genomic pathogen surveillance. Usability, speed, and security are additional requirements for practical outbreak analysis in a healthcare setting.

Our evaluation demonstrates that the Solu platform produces outputs that are largely consistent with prior outbreak studies, using raw sequencing reads and requiring zero configuration, with a runtime of approximately 10 min for bacterial samples and 20 min for fungal samples. Solu’s phylogenetic pipelines produced results that were internally and externally consistent.

The largest discrepancies were observed in the *Staphylococcus aureus* dataset, where the original study used PCR for MLST assignment and applied 90% identity and 75% coverage thresholds for AMR gene detection. We hypothesize that the different pipeline parameters allow for higher sensitivity, at the cost of potential misidentification.

Compared to some other pipelines, Solu platform’s zero-configuration design prevents users from customizing pipeline parameters, which may result in some variation in the results. This approach was chosen to promote usability and prevent users from inadvertently selecting unsuitable parameters. Despite this limitation, default tool configurations provide sufficient accuracy for a wide variety of research applications, including AMR gene characterization and clonality assessment [46]. Future studies leveraging more in-depth datasets and epidemiologically validated outbreaks hold great potential to further strengthen and expand the applicability of our findings.

We aim to improve the analytical capacity of the platform in future iterations, featuring additional tooling, modifications to the analytical workflow, broader support for species and databases, and improved runtimes among other features. We encourage users to contact the authors to request any additional analyses or databases of interest.

In conclusion, by focusing on a robust, privacy-focused infrastructure, Solu facilitates broader adoption of genomic pathogen surveillance, potentially bridging the gap between research and practice.

## Supplementary Information

**Additional file 1.** Bioinformatics pipeline description.

**Additional file 2.** Additional phylogenetic pipeline evaluation results.

## Data Availability

The data analyzed in the study are available from the European Nucleotide Archive at https://www.ebi.ac.uk/ena using the accessions provided in Table 1. The Solu workspace, including all samples and results, is accessible at https://platform.solugenomics.com/w/solu-publication.

## Abbreviations

AFR: Antifungal resistance
AMR: Antimicrobial resistance
CPU: Central Processing Unit
CT: Complex type
FAIR: Findable, Accessible, Interoperable, Reusable
HIPAA: Health Insurance Portability and Accountability Act
MLST: Multi-locus Sequence Typing
QA: Quality assurance
SNP: Single nucleotide polymorphism
ST: Sequence type
WGS: Whole-Genome Sequencing
