# Supplementary material for: Solu: a cloud platform for real-time genomic pathogen surveillance
Source: BMC Bioinformatics. 2025 Jan 13;26:12. doi: 10.1186/s12859-024-06005-z (PMC11731562; doi:10.1186/s12859-024-06005-z)
Supplement: Supplementary file 2 — Additional file 2. Additional phylogenetic pipeline evaluation results. [file 12859_2024_6005_MOESM2_ESM.pdf]

S2. Additional phylogenetics results

Figure 1. Solu's phylogram of the S. aureus dataset vs. reported sequence types (outer ring)

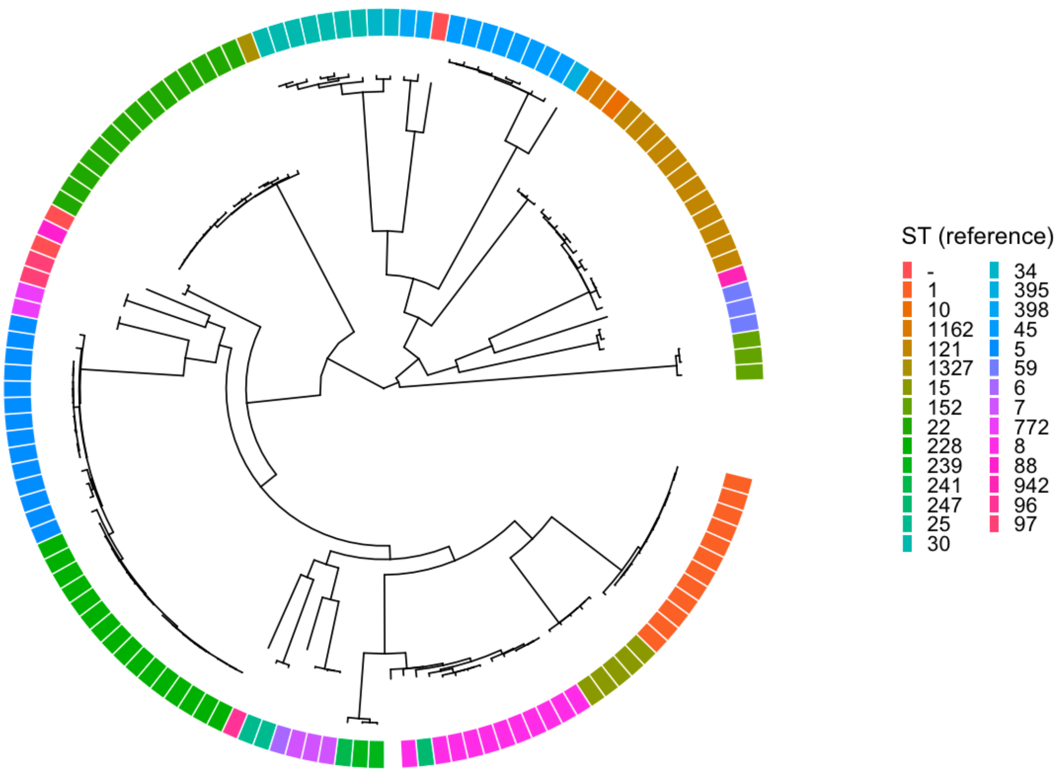

Figure 2. Solu's phylogram of the Candida auris dataset vs. reported clade (outer ring)

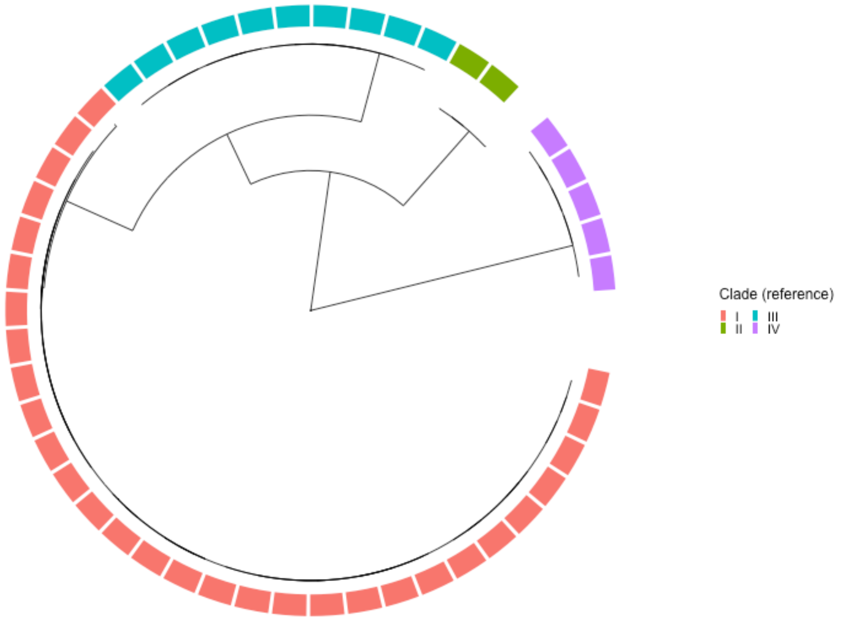

Table 1. Validation of Solu's reference-based vs. reference-free phylogenetics

**Dataset**	**Robinson-Foulds distance of Solu's reference-based tree vs. Solu's reference-free tree**
S. aureus	0.08093671
E. faecium	0.2694115

S. enterica	0.4644507
C. auris	0.2362036

The tree distances were calculated with TreeDist 2.9.1 (1).

Figure 3. Tanglegram of *Staphylococcus aureus* reference-free (left) vs. reference-based (right) phylogenetic trees

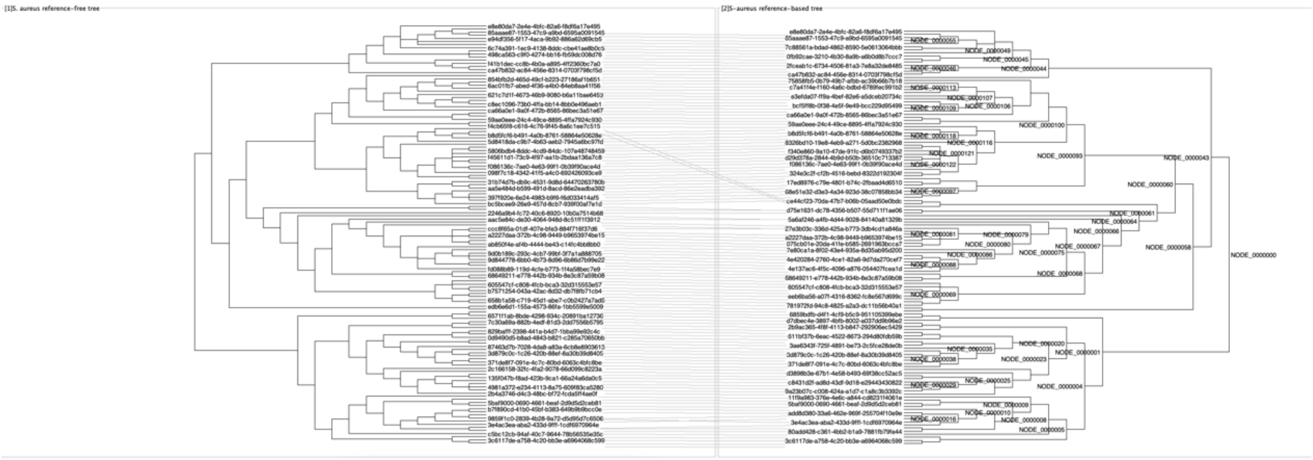

Figure 4. Tanglegram of *Salmonella enterica* reference-free (left) vs. reference-based (right) phylogenetic trees

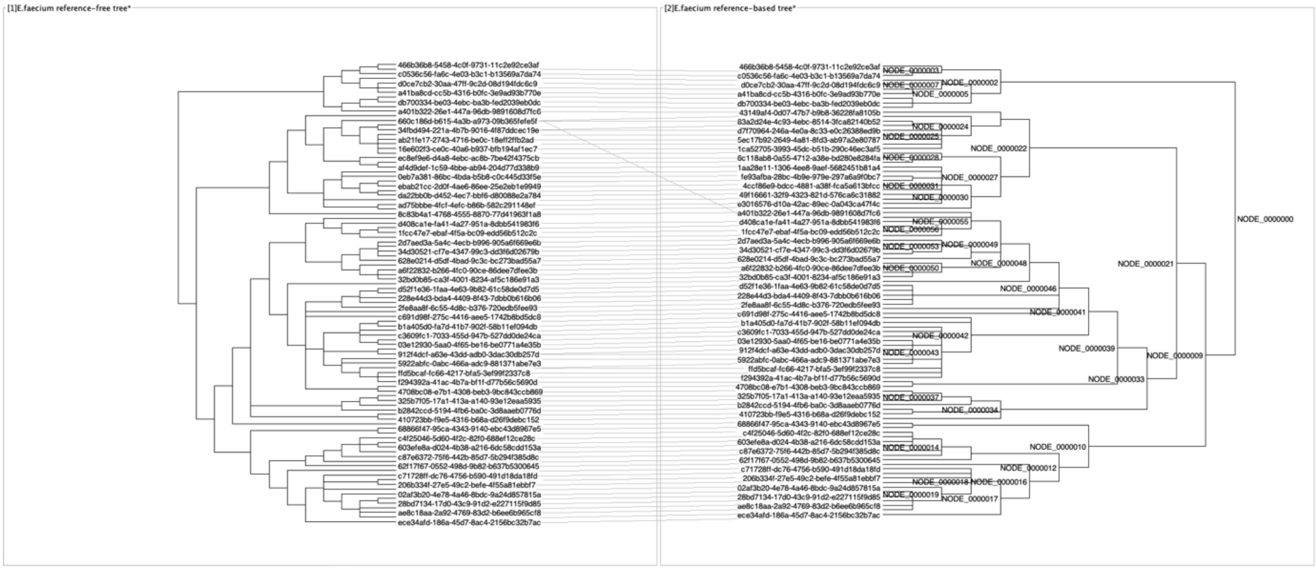

Figure 5. Tanglegram of *Candida auris* reference-free (left) vs. reference-based (right) phylogenetic trees

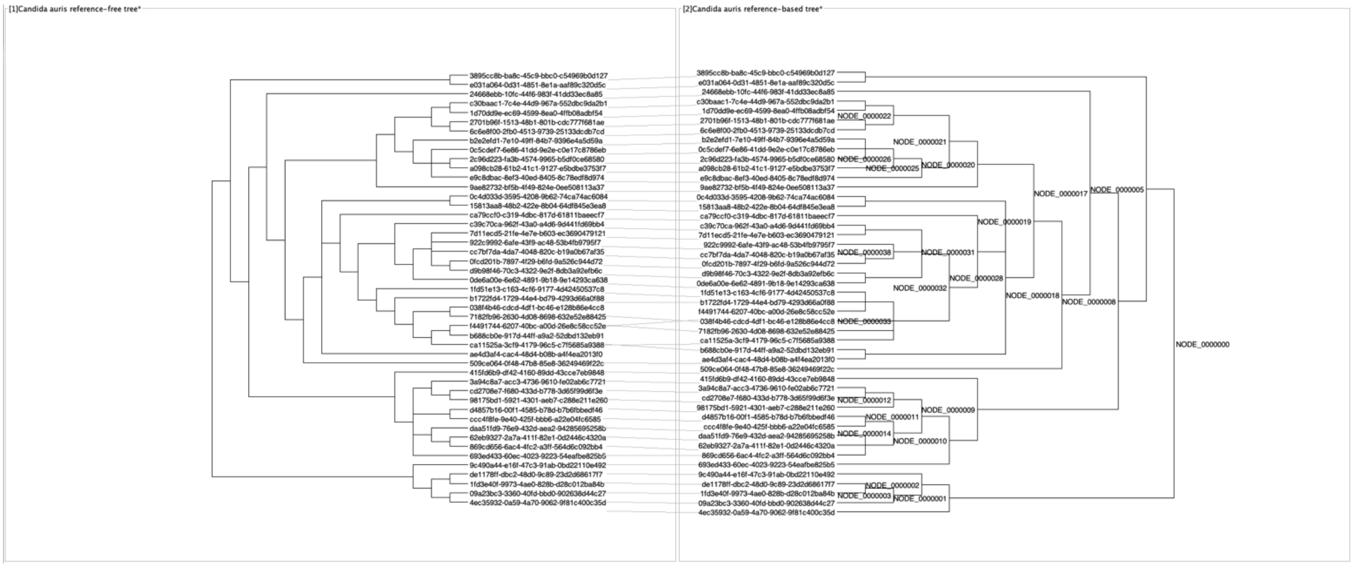

Figure 6. Tanglegram of *Salmonella enterica* reference-free (left) vs. reference-based (right) phylogenetic trees

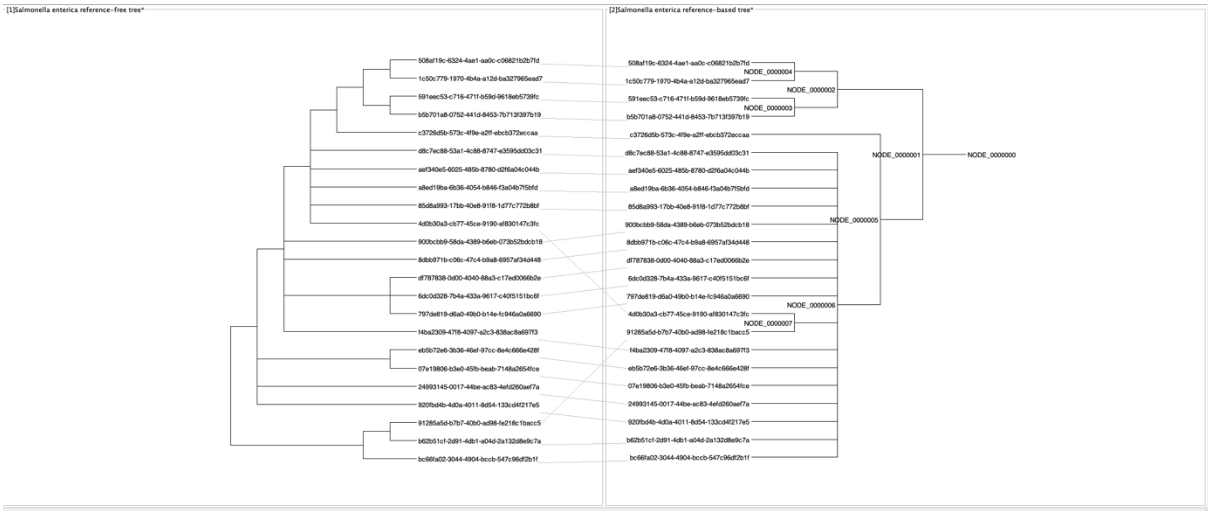

All tanglegrams were created with Dendroscope v 3.8.10 (2).

References

1. Smith MR. Information theoretic generalized Robinson–Foulds metrics for comparing phylogenetic trees. 2020;36(20):5007–13.

2. Huson DH, Scornavacca C. Dendroscope 3: An Interactive Tool for Rooted Phylogenetic Trees and Networks. Syst Biol. 2012 Dec 1;61(6):1061–7.
